# Mandibular radiomorphometric assessment of bone mineral density in survivors of pediatric hematopoietic stem-cell transplantation

**DOI:** 10.6061/clinics/2019/e929

**Published:** 2019-05-22

**Authors:** Alexandre Viana Frascino, Claudio Costa, Daniela Miranda Richarte de Andrade Salgado, Fabio Luiz Coracin, Marcelo Fava, Vicente Odone-Filho

**Affiliations:** I Departamento de Pediatria, Faculdade de Medicina FMUSP, Universidade de Sao Paulo, São Paulo, SP, BR; II Departamento de Estomatologia, Faculdade de Odontologia, Universidade de Sao Paulo, Sao Paulo, SP, BR; III Departamento de Saude, Faculdade de Odontologia, Universidade Nove de Julho, Sao Paulo, SP, BR; IV Odontologia Pediatrica, Faculdade de Odontologia, Universidade Estadual Paulista (UNESP), Sao Jose dos Campos, SP, BR

**Keywords:** Bone Marrow Transplantation, Hematopoietic Stem Cell Transplantation, Children, Osteoporosis, Radiomorphometric Assessment

## Abstract

**OBJECTIVE:**

Hematopoietic stem-cell transplantation (HSCT) childhood survivors of hematologic malignancies are prone to develop late osteopenia and osteoporosis. The purpose of this retrospective study was to quantitatively and qualitatively assess bone mineral density (BMD) in HSCT childhood survivors and to compare the effectiveness of both qualitative and quantitative assessment methods.

**METHODS:**

DESIGN BMD assessment using panoramic radiographs of childhood HSCT survivors aged 3.69-18.88 years using two radiomorphometric indexes. Case-control double-blinded comparison of panoramic radiographic images from childhood HSCT survivors and age- and sex-matched healthy controls. Quantitative assessment was performed by measuring the cortical bone width bilaterally at the mental foramen level. Qualitative assessment was performed using the mandibular cortical index bilaterally on all panoramic images.

**RESULTS:**

Radiographs were taken 6.59-83.95 months after bone marrow transplantation [median±SD=25.92±24.9 months]. Fifty-two panoramic radiographic images were analyzed: 21 from HSCT survivors and 31 from healthy controls aged 3.69-25.1 years [mean±SD=11.89±5.28 years]. The mandibular cortical bone width was 17% smaller in childhood HSCT survivors than in healthy controls (case group: 2.420, control group: 3.307; *p*=0.00617). Qualitative analysis revealed an increased frequency of severe mandibular cortical erosion in childhood HSCT survivors, although no significant difference was observed (case group: 1.540, control group: 1.490; *p*=0.32). The interobserver agreement was 85% (Kappa index).

**CONCLUSIONS:**

HSCT childhood survivors exhibit quantitative and qualitative mandibular bone impairments. Further studies are needed to establish an association between mandibular cortical bone impairment and osteoporosis.

## INTRODUCTION

Hematopoietic stem-cell transplantation (HSCT) plays an important role in the curative management of hematologic high-risk pediatric malignancies, with high rates of long-term disease-free survival 1,2. Childhood HSCT survivors may be substantially affected by a wide range of adverse late effects, including reduced bone mineral density (BMD) and increased risk of bone fractures, which negatively impact their overall quality of life 3-5.

Dual-energy X-ray absorptiometry (DXA) of long bones or vertebrae is the preferred method for the diagnosis of low BMD. Despite its high cost, DXA is well established as the gold standard examination for diagnosing osteopenia/osteoporosis, as recommended by the World Health Organization (WHO) 6.

Morphometric evaluation of panoramic jaw radiographs has been proposed as an alternative to DXA for the screening, diagnosis, and evaluation of low BMD 7,8. The qualitative and quantitative assessment of mandibular cortical bone provides predictive information for the early diagnosis of osteoporosis in adults 9-12, but few studies have been conducted in children 13.

We hypothesized that childhood HSCT survivors have reduced BMD compared to that of healthy control individuals. Using panoramic radiographs, this study aimed to assess bone health quantitatively and qualitatively in childhood HSCT survivors and in age- and gender-matched controls and to compare the effectiveness of both qualitative and quantitative assessment methods.

## METHODS

This study was approved by the Research Ethics Committee of the University of São Paulo Medical School (CEP/CONEP number 1.034.483/2015). The case (HSCT) group consisted of digital panoramic radiographic images from childhood HSCT survivors aged 3.69 to 18.88 years who were recruited at the Pediatric Oncology Institute, University of São Paulo Medical School (ITACI-ICR/FM USP), São Paulo, Brazil. The inclusion criteria were as follows: (1) HSCT for the treatment of hematological malignancies; (2) age at time of HSCT ≤18 years; and (3) panoramic radiograph taken at least six months after HSCT.

Information including medical history, birth date, gender, location, age at cancer diagnosis, age at HSCT, and myeloablative conditioning regimen was collected from medical records. Information about the primary malignancy diagnosis was obtained from primary caretakers, and the dental history was retrieved from dental records at the Dental Clinic of the Children's Institute at the University of São Paulo Medical School.

Panoramic radiographs were taken by the same operator at the Radiology Institute (InRad) of the University of São Paulo Medical School using the same X-ray panoramic machine (Orthophos CD, Siemens, Bensheim, Germany) with imaging settings of 60-90 kVp, 9-12 mAs, and 12 s of exposure. All radiographic images were downloaded with 256 gray levels, 3188×1709 pixels, and 300 dpi resolution in digital format (JPEG) compatible with ImageJ (1.50c4 for Mac OS Sierra 10.12.6, available at https://imagej.nih.gov/ij/).

The control group (CONTROL) consisted of age- and gender-matched healthy subjects selected from a databank at the Dentistry School of the São Paulo State University in São José dos Campos (UNESP-SJC), São Paulo. The exclusion criteria were as follows: (1) early medical complications or previously diagnosed developmental skeletal disorders; (2) previous orthodontic treatment; and (3) history of craniofacial trauma. All participants' parents or legal guardians provided written consent for their children to participate in the study and were free to withdraw their participation at any time.

### Quantitative Assessment

Quantitative analyses of panoramic images were performed using ImageJ (1.50c4 for Mac OS Sierra 10.12.6). The morphometric analysis of radiographic images was performed using well-established radiographic assessment methods proposed by Taguchi et al. (2004) 10. The images were analyzed independently by two blinded researchers, an experienced oral radiologist and an oral and maxillofacial surgeon with 10 years of experience. The images were viewed using the same computer monitor in a quiet, dimly lit room.

**Figure 1:**
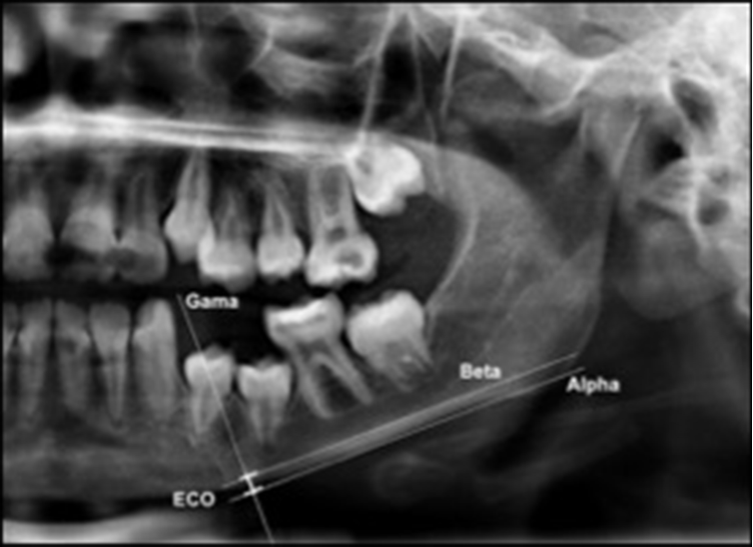
Mandibular cortical width as proposed by Taguchi et al. (2004) (10). Alpha: external lamina of the mandibular basal line; Beta: internal lamina of the mandibular basal line; Gamma: perpendicular intersection at the mental foramen level. ECO: bone width of the inferior mandibular cortex.

The mandibular cortex width was measured bilaterally at the mental foramen level using lines parallel to both the external and internal cortical laminae of the mandibular basal line (Alpha and Beta, respectively). Perpendicular to these lines, an intersecting line was drawn diametrically dividing the mental foramen (Gamma). The distance between the two parallel lines at the intersection with the perpendicular line (ECO) was considered the cortical width of the mandibular inferior border (Figure 1).

### Qualitative Assessment

The width of the inferior mandibular cortex measured bilaterally in the premolar region in both groups was analyzed by two experienced blinded observers, and interobserver agreement was calculated using the Kappa index. The images were viewed separately by each observer using the same computer monitor in a quiet, dimly lit room.

Panoramic radiographic images were categorized according to the Klemetti index 9,11-14 as shown briefly in Table 1.

### Statistical Analysis

The hypothesis of quantitative mandibular bone changes in childhood HSCT survivors compared with age- and gender-matched healthy controls was tested using the paired *t*-test in Excel for Mac (Microsoft version 15.37).

The hypothesis of qualitative mandibular bone alterations was tested using the chi-square test in Excel for Mac (Microsoft version 15.37). All other qualitative or categorical variables were described as frequencies and proportions.

## RESULTS

### Patients and treatment characteristics

Fifty-two panoramic radiographic images were analyzed: 21 from childhood HSCT survivors (14 males and 7 females) and 31 from healthy controls (16 males and 15 females). Participant ages ranged from 3.69 to 25.1 years [mean age±SD=11.89±5.28 years], and there was no significant difference in mean age between cases and controls (*p*=0.88). The age distribution of both groups is shown in Table 2.

**Figure 2:**
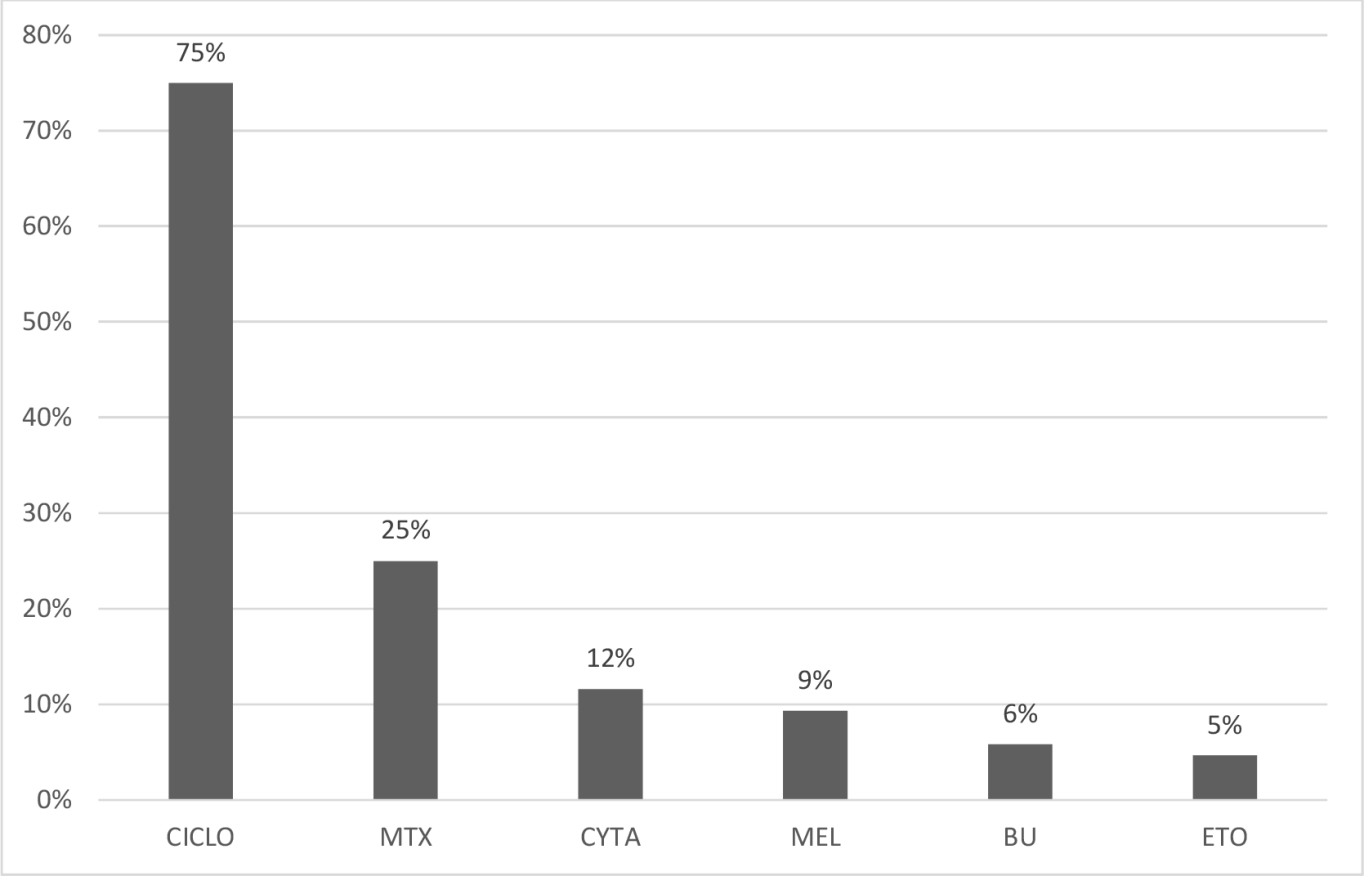
Myeloablative chemotherapy drug distribution. CYCLO: cyclophosphamide; MTX: methotrexate; CYTA: cytarabine; MEL: melphalan; BU: busulphan; ETO: etoposide.

The primary malignancy diagnosis was acute myeloid leukemia (56%), acute lymphoblastic leukemia (38%), and myelodysplastic syndrome (6%). The HSCT type was autogenous in 29% of patients, allogenic in 58% of patients, and umbilical cord in 21% of cases. Myeloablative conditioning was achieved by individualized drug schemes associated with total body radiotherapy in 75% of cases. Cyclophosphamide was administered in 75% of cases in association with other drugs (Figure 2).

Radiographic images from the HSCT patients were obtained 6.59-83.95 months after bone marrow transplantation [median±SD=25.92±24.9 months] between September 2015 and December 2017.

### Quantitative Analyses

The interobserver agreement of quantitative analyses was 85%. The mandibular cortical bone width was 17% smaller in HSCT patients than in healthy controls (case group: 2.420, control group: 3.307; *p*=0.00617). Dispersion analyses indicated a narrower distribution in the case group (minimum: 1.028; maximum: 3.498; median: 2.605).

### Qualitative Analyses

**Figure 3:**
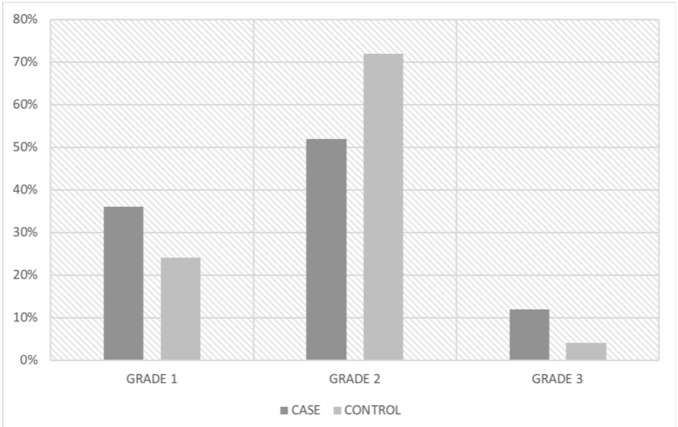
Qualitative assessment of mandibular cortical bone erosion in childhood HSCT patients and healthy controls by the Klemetti index (%).

The interobserver agreement of the qualitative analyses was 85%. The qualitative analysis revealed a higher prevalence of severe cortical erosion in the case group, though no statistical significance was found (case group: 1.540, control group: 1.490; *p*=0.32) (Figure 3).

## DISCUSSION

This study shows that compared to age- and gender-matched healthy controls, childhood HSCT survivors (3.69-18.88 years old) of hematologic malignancies have both quantitative and qualitative mandibular cortical bone impairments. These results agree with previous investigations that show that reduced BMD is significantly associated with survivor rates 1,2.

Quantitative bone changes in these patients are often described in terms of femoral and lumbar bone deficits and reduced BMD 4. HSCT involves corticosteroid administration, which inhibits multiple pathways, including reduced osteoblastic activity, increased osteoclastic bone resorption, and inhibition of vitamin D 1α-hydroxylation with impairment of intestinal calcium absorption and reduced muscle strength 18. Additionally, endocrine complications due to chemotherapy and/or radiotherapy, such as growth hormone deficiency and hypogonadism, can adversely affect BMD 19.

The qualitative skeletal changes in young adult survivors of childhood HSCT are associated with an increased risk of bone fractures 13,20. In a large-scale study, Pundole et al. (2015) reported that HSCT recipients were approximately 7-9 times more likely to develop fractures than the general population in the United States 21. Although bone maturation during puberty increases BMD, children exposed to chemotherapy early in life show reduced BMD at older ages 3. The increased risk of fractures negatively impacts quality of life and may ultimately result in extended periods of intensive level medical care and, potentially, life-sustaining treatment modalities 22.

The quantitative and qualitative evaluation methods for BMD impairment used in this study are well-documented techniques that enable the classification of radiographic changes in the mandibular cortex in a cost-effective and highly reproducible manner. Mandibular cortical width has been shown to be positively associated with BMD 10. The Klemetti index is a well-established screening method for reduced skeletal BMD in adults and elderlies 12,14,23. However, Allen et al. (2016) found no association between qualitative changes in the mandibular cortex and BMD deficits in children and young adult survivors of acute lymphoblastic leukemia (ALL) 13.

The current study supports previous research showing that dentists are able to identify patients at risk of low BMD based on mandibular cortical erosion on panoramic radiographs and refer such patients to further exams 10,23. Dental practitioners are part of the healthcare team and can actively help in the early diagnosis of low BMD. Panoramic radiographs are a routine part of the dental evaluation by general dentists, orthodontists, and oral and maxillofacial surgeons; they are a low-cost, easy to perform, standardized exam that exposes patients to low doses of ionizing radiation 24 and could serve as a screening tool to prevent late effects of HSCT in adult life through highly sensitive exams that can ultimately help improve multidisciplinary care and quality of life 25.

One methodological limitation of the present study was the absence of more extensive follow-up of childhood HSCT survivors. The study population was recruited at the Pediatric Oncology Institute of the University of São Paulo Medical School, a public tertiary reference center for multiple regional public health care units that provides diagnosis for more complicated cases frequently associated with higher recurrence and lower survival rates. In this scenario, antineoplastic regimens for myeloablative purposes are highly tailored with multiple drug schemes and precise dose adjustments, resulting in nonhomogeneous study groups.

In summary, compared to healthy individuals, childhood HSCT survivors exhibit significant quantitative changes in mandibular cortex width with an increased risk of fractures. Further studies are needed to assess the risk of osteoporosis in childhood HSCT survivors.

## CLINICAL IMPLICATIONS

- This paper identifies a deficit where oral healthcare providers could improve the overall health in children after HSCT.
- This paper demonstrates that the Klemetti technique is not a predictor of BMD in this population but that Taguchi's evaluation method could be used as a screening protocol.
- Childhood HSCT survivors are a population at risk for reduced BMD, and close follow-up is needed.

## AUTHOR CONTRIBUTIONS

Frascino AV and Costa C conceived the ideas. Fava M and Salgado DMRA collected the data. Coracin FL analyzed the data. Odone-Filho V led the writing of this manuscript.

## Figures and Tables

**Table 1:** Klemetti index categories for panoramic radiographs.

**Grade 1** Normal cortex	The endosteal margin shape is even and sharp. The inner cortical layer is uniform, and there are no bone excavations.	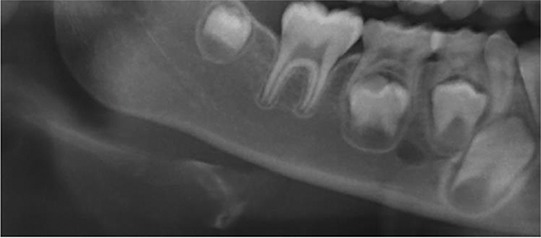
**Grade 2** Mild to moderate erosion	The endosteal margin shows few semilunar-shaped bone resorptions and/or irregularities within the inner cortex.	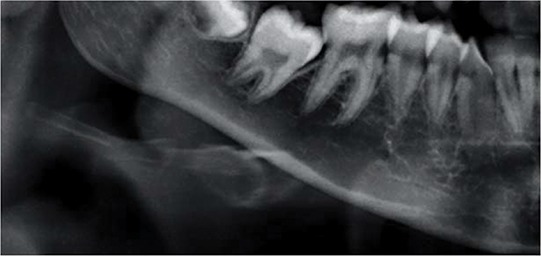
**Grade 3** Severe cortical erosion	The cortical layer forms deep cortical concavities and is clearly porous. The inner cortex shows several irregularities.	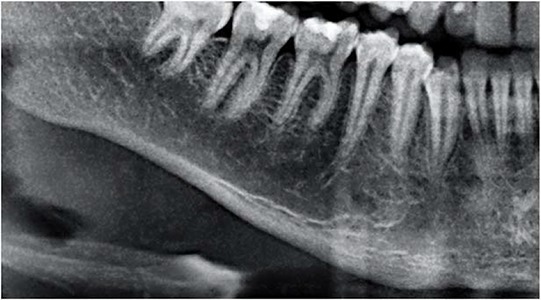

**Table 2:** Age (years) distribution of childhood HSCT patients and matched controls.

	HSCT	Control	Total
Youngest	3.69	4.69	3.69
Oldest	18.88	25.1	25.1
Mean age	11.76	11.98	11.89
SD	4.34	5.9	5.28

SD: standard deviation.
